# The Relationship between Technology Use and Physical Activity among Typically-Developing Children

**DOI:** 10.3390/healthcare8040488

**Published:** 2020-11-17

**Authors:** Thekra Alotaibi, Rifan Almuhanna, Johara Alhassan, Ethar Alqadhib, Eman Mortada, Reem Alwhaibi

**Affiliations:** 1 Rehabilitation Sciences Department, College of Health and Rehabilitation Sciences, Princess Nourah Bint Abdulrahman University, Riyadh 11466, Saudi Arabia; thekra_alotaibi@hotmail.com (T.A.); Rifan.Almuhanna@gmail.com (R.A.); johara-alhassan@hotmail.com (J.A.); ethar.m.q@gmail.com (E.A.); 2 Health Sciences Department, College of Health and Rehabilitation Sciences, Princess Nourah Bint Abdulrahman University, Riyadh 11466, Saudi Arabia; EMMortada@pnu.edu.sa

**Keywords:** physical activity, youth, technology use, parents’ questionnaire, children

## Abstract

Objectives: This study aimed to investigate the relationship between technology use and physical activity level and to measure the association between sociodemographic characteristics of the participants, technology use, and physical activity level among Saudi children. Materials and Methods: This cross-sectional study was conducted among 458 parents of typically-developing Saudi children (6–12 years). A translated validated questionnaire used for data collection consisted of three parts: Children’s Physical Activity Questionnaire (CPAQ), Questionnaire on the Impact of Technology on Children (used to investigate the impact of technology on children’s physical activity) and sociodemographic questions (e.g, children’s age and sex, age, educational level, marital status of parents and monthly income). Data were analyzed using Pearson correlation and Mann-Whitney U test to assess the relationship between technology use and physical activity level. A chi-squared test was used to assess the relationship between technology use and sociodemographic variables. Statistical significance was set at *p* < 0.05. Results: Mean age of the sampled children was (8.44 ± 2.07). Data analysis revealed that high use of technology was significantly associated with low level of activity. Pearson’s correlation analysis showed a negative relationship between a high level of activity and technology use (*r* = −0.138, *p* = 0.047). Ownership of a device was significantly associated with higher technology time consumption. Regression analysis revealed that age of the child, educational level of the parents, screen time use, and owning electrical devices significantly predicted the level of practicing physical activity among children of sampled parents (*p* < 0.05). Conclusions: practicing inadequate physical activity among children could be influenced by educational level of parents, screen time use, and owning electrical devices. Therefore, parental involvement is required to reduce time of exposure to technology screens among children.

## 1. Introduction

Children are increasingly attracted to the newest forms of technology [1]. With the recent rise in popularity of smart phones and tablets, the use of such technological devices has become unavoidable and is now viewed as an integral part of life. According to the General Authority of Statistics (GAS) in Saudi Arabia, 98.44%, 61.08%, 83.87%, and 47.21% of households or families have televisions, use computers, are internet users, or own smart devices, respectively [2].

Technology use is associated with increased risk of obesity in children [3], type 2 diabetes mellitus, and all-cause mortality [4]. It has also been associated with increased risk of metabolic syndrome [5], and a large range of physical and psychological disorders seen in technology-dependent children [6]. Technology-dependent children tend to have impaired quality of life and are often affected emotionally, socially, and academically [7,8]. Increased dependency on technology was associated with increased level of anxiety symptoms, depression, aggression, and attention and behavioral problems [9]. It was also associated with lower academic performance, Grade Point Average (GPA), language, math and reading achievements [9,10]. Furthermore, increased screen-time use reduces children’s opportunities to interact and socialize with others, thereby affecting the normal development of healthy social skills [11].

Because technology use interrupts the daily activities of children, it can result in decreased physical activity. Approximately 3.2 million deaths per year globally are related to physical inactivity; it is thus a risk factor for high mortality. Physical activity is essential to stabilize blood pressure and glucose levels, maintain normal body weight, improve sleep patterns, and improve immune response and metabolism [12]. According to the World Health Organization (WHO), the developing child needs at least one hour per day of moderate to vigorous intensity physical activity [12]. The benefit is higher if the daily activity exceeds 60 min [12].

Factors that limit children’s physical activity have been previously investigated. Brustad [13] found that children’s attraction to physical activity is strongly related to social and psychological variables. Sex, parental socialization, and a child’s self-perception characteristics may affect their physical activity behaviors [13]. Maturo & Cunningham [14] discussed the influence of friendship in promoting lifelong healthy habits in children. Low-income families often have limited financial resources to afford physical activity opportunities for their children and working parents have limited time to interact during physical activity with their children. Figaji and Philips [15] recommend that parents, teachers, and friends should support children’s participation in physical activity, especially since they spend a considerable time at school.

Technology use among children is one of the predicting factors for physical inactivity. Kenney and Gortmaker [16] found that excessive use of smartphones, tablets, computers, and videogames was positively related to a lack of physical activity. Moreover, television time was found to be a factor limiting a child’s physical activity time [17]. One study conducted on five-year-old children showed that those with high technology use are less active and more likely to have sedentary lifestyles [18]. Recently, Webster et al. [19] showed positive associations between fundamental motor skills and vigorous physical activity among preschoolers, but inverse associations with screen-time.

To our knowledge, studies investigating the relationship between technology use and physical activity among typically-developing children have been conducted in North America [16,19,20,21,22,23], Europe [3,6,17,18,24,25,26,27,28,29], Australia [4,14,30,31], and in a few countries in Asia [32,33,34] and Africa [15]. The only study that was conducted in Saudi Arabia was in 2014 and with adolescents aged between 15 and 19 years of age to investigate the association of dietary habits with physical activity and technology use [35]. Therefore, the objectives of this study were to investigate the relationship between technology use and physical activity among typically-developing Saudi children aged between 6–12 years, and to measure the association between sociodemographic characteristics, technology use, and physical activity level.

## 2. Materials and Methods

### 2.1. Design

A cross sectional study was conducted to investigate the relationship between technology use and physical activity level among typically-developing Saudi Arabian children using an online-questionnaire survey. Physical activity is defined as “any bodily movement produced by skeletal muscles that requires energy expenditure” [8]. Participants in the study included parents of typically-developing children between the ages of 6–12 years. Participants who identified themselves as parents of typically-developing children between the ages of 6–12 years within Saudi Arabia and who agreed to participate in the study were eligible. This study was approved in advance by the Institutional Review Board (IRB) of Princess Nourah bint Abdulrahman University (17-0197). An anonymous online approach was adopted and a statement in the introduction of the survey clarified that participation was entirely voluntary, participants could withdraw at any time, and privacy and confidentiality of participant information would be completely protected. Participants were recruited via convenience and snowball sampling by word-of-mouth and referral strategies. A calculated sample of 400 parents would provide a margin of error of 5% from the true values at a 95% confidence level. Consequently, the total number of the respondents was increased by 14% to obtain a sample of 458, accounting for nonresponse.

The datasets used and/or analyzed during the current study are available from the corresponding author on reasonable request.

### 2.2. Procedure and Tools

An anonymized survey was distributed using an online platform to recruit parents of typically-developing Saudi Arabian children. Parents completed the survey on demographics, electronics use and physical activity in the Arabic language. The survey took approximately 8 to 10 min to be completed by the parents. Incomplete and duplicate questionnaires were discarded.

The survey consisted of three parts: demographic data, Children’s Physical Activity Questionnaire (CPAQ) [36] and The Impact of Technology on Children [7].

#### 2.2.1. Part I: Demographic Data

A personal information questionnaire prepared by the researchers was used to gather sociodemographic information. It was divided into three parts. The first section covered information related to the parents (relation to the child (mother, father), age, educational level, and marital status). The second section covered information related to the child (age and sex). The third section covered information regarding the family (family monthly income, number of children, district).

#### 2.2.2. Part II: Children’s Physical Activity Questionnaire (CPAQ)

Parents were required to assess mode, frequency, and duration of physical activity and sedentary activities, including school time and leisure time, over the past seven days. The response options were indicated by “yes” or “no” and by indicating the total time spent on the activity during the week (hours per week). The first section of the questionnaire assessed activities performed during the past seven days, including soccer, volleyball, tennis, swimming, basketball, and running. The second section evaluated leisure time activities including bike riding, household chores, playing with pets, bouncing on the trampoline, scooter, playing on playground equipment, walking, or skipping rope. Total non-school sedentary time was also calculated from the time reported in the questionnaire, and included time for activities such as arts and crafts, homework, listening to music, traveling in the car/bus, reading, and playing board games/cards. Total screen-time was also calculated and included all time spent using computer/internet/devices, watching television/videos, and playing computer games during the day, outside school hours [37]. The validity and reliability of the test have been reported in several studies and, although study designs and populations have been varied, overall results have been convincing. The questionnaire showed high reliability ranging from 0.78 to 0.94, and moderate validity ranging from 0.63 to 0.71 [38,39,40,41,42].

#### 2.2.3. Part III: The Impact of Technology on Children

The Impact of Technology on Children questionnaire [7] was developed by Dr. Jacqui Taylor from Bournemouth University, Psychology Research Centre, Talbot Campus to assess the impact of technology on children. This questionnaire is suitable for use in children aged 4–12. It contains questions regarding the child’s use of technology, after-school activities, sleep patterns, behavior, and emotions. For the purpose of the current study and to avoid repetition, only the questions related to the child’s use of technology were used. This part of the survey included questions about the child’s ownership of electronic devices, number and type/s of device/s owned by the child, age when the child first owned a device, number of hours/day spent watching television or DVDs, playing video games such as PS3^®^ (Sony Corporation, Tokyo, Japan), Xbox^®^ (Microsoft, Redmond, WA, USA), or Wii^®^ (Foxconn, New Taipei City, Taiwan) or playing internet games, playing on a portable games console, e.g., iPad^®^ (Foxconn, New Taipei City, Taiwan), iPod^®^ (Apple Inc., Cupertino, CA, USA), PSP^®^ (Sony Corporation, Tokyo, Japan), or Nintendo DS^®^ (Foxconn, New Taipei City, Taiwan), and use of Facebook or other social Networking sites, e.g., Twitter (Twitter Inc., San Francisco, CA, USA) or Instagram (Facebook Inc., Menlo Park, CA, USA).

### 2.3. Translation of the Questionnaires

CPAQ and the Research Questionnaire on the Impact of Technology on Children were translated into Arabic and validated before use, using a standardized forward and backward translation procedure, as recommended by Bradley [43]. Initial translation of the questionnaires from English to Arabic was performed by a panel of three health experts who speak both languages fluently. Another bilingual health professional, blinded to the English version, backward-translated the questionnaires into English. A certified simultaneous Arabic-English interpreter assessed the backward-translated English version of both questionnaires to maintain the semantics and meanings of the English versions. Using the first-translated questionnaires, a pilot test was conducted among 10 participants. For this pilot test, the questionnaires were self-administered. A face-to-face interview was then performed by the researcher to ask the participants whether any items were confusing or difficult to answer. The first-translated questionnaires were then revised accordingly by the two initial translators to develop the final Arabic version, considering the feedback and proposed changes based on the pilot test. The final Arabic version was re-pilot tested among another five participants to confirm that the instructions, questions, and response options could be clearly understood.

### 2.4. Data Analysis

Statistical analysis was performed using IBM SPSS Statistics for Windows, Version 20.0 (IBM Corp., Armonk, NY, USA). Descriptive statistics were used to present the general characteristics of the participants using frequencies and percentages for categorical variables and means and standard deviations for quantitative variables. Pearson correlation and a Mann-Whitney U test were used to assess the relationship between technology use and physical activity level among typically-developing children. A chi-squared test was used to assess the relationship between technology use and sociodemographic variables. Statistical significance was set at *p* < 0.05. The analysis of the Research Questionnaire on the Impact of Technology on Children was as follows.

Technology consumption time was dichotomized for statistical purposes using median as a cutoff point as follows: total hours spent on the internet, social media, video games, and portable devices was calculated then subjects were divided into two groups. The first and second groups included children who spent 5 h or less (screen-time ≤5 h), the low screen-time users, while those using screens for 6 h or more (screen-time ≥6 h) are categorized as high screen-time users.

For the CPAQ, physical activities were classified depending on the metabolic equivalent of task (MET) into three categories: light-intensity (MET < 3), moderate-intensity (MET 3 to 6), and vigorous-intensity (MET > 6). The physical activities’ MET values were obtained from the Compendium of Physical Activities; the updated version of activity codes and MET intensities were used [44]. Accordingly, each child’s physical activity hours were calculated during the week and divided, based on each MET. The highest number of hours in each category was recorded as the child’s activity level.

## 3. Results

### 3.1. Sample Characteristics

After 52 responses were excluded because of incomplete questionnaires, 458 parents responded to the survey. The results of the demographic characteristics of the study population showed that 86.5% of responses were from mothers (*n =* 396) and 13.5% from fathers (*n =* 62). The highest response was from parents aged 30–35 years (*n =* 256; 55.9%), followed by those aged 35–40 (*n =* 139; 30.3%), 25–30 (*n =* 44; 9.6%), and the lowest from those >40 (*n =* 19; 4.1%).

Most respondents were married (*n =* 418; 91.3%). Only 3.7% were divorced (*n =* 17), 1.5% separated (*n =* 7), and 3.5% widowed (*n =* 16). The majority of respondents (*n =* 317; 69.2%) had a bachelor’s degree, while 11.4% were high school graduates (*n =* 52), 17.2% were postgraduates (*n =* 79), 1.7% had less than high school degree (*n =* 8), and only 2 (0.4%) were not educated.

The monthly income of most respondents ranged from 10,000 to 15,000 SAR ($2600–$4000) (*n =* 151; 33%), followed by those who earned more than 20,000 SAR ($5000) (*n =* 124; 27.1%), those earning between 15,000–20,000 SAR ($4000–$5000)(*n =* 87; 19%), from 5000–10000 SAR ($1300–$2600) (*n =* 74; 16.2%), and less than 5000 SAR ($2600) (*n =* 22; 4.6%). The children of parents from whom the data were collected were aged between 6–12 years (mean age; 8.44 ± 2.07 years), consisting of more boys (*n =* 244; 53.3%) than girls (*n =* 214; 46.7%).

### 3.2. Technology Use Results

Table 1 shows a statistically significant association between screen-time use in children and marital status, family income, child’s age, and child’s ownership of device (*p* < 0.05). Conversely, there was no significant association between screen-time use and educational level, parental age, sex of the child, and the child’s age when he/she owned their first device.

### 3.3. Physical Activity and Technology Use Results

Table 2 shows a significantly negative correlation between hours of technology use (television, social media, video games, internet, and portable devices) and a high level of physical activity (*r* = −0.138; *p* = 0.047). Conversely, there was weak negative correlation between technology use and moderate physical activity; however, it was not significant (*r* = −0.130; *p* = 0.062). There was a positively weak, insignificant correlation between technology use and low physical activity (*r* = 0.136; *p* = 0.051).

Regression analysis revealed that age of the child, educational level of the parents, screen time use, and owning electrical devices significantly predicted the practice level of physical activity among children of sampled parents (*p* < 0.05) (Table 3).

## 4. Discussion

### 4.1. Correlation Between Technology Use and Physical Activity

The present study found that hours of technology use including television, social media, video games, internet, and portable devices had a significantly negative correlation with a high level of physical activity. Our findings are consistent with those of previous studies; Serrano-Sanchez et al. [29] and Motamed-Gorji et al. [45] found that the more screen-time spent, the less physical activity the child performs. Rideout et al. [46] suggested that youths can spend up to 11 h per day using screen-based technologies, which can be associated with snacking and excessive eating, potentially replacing participation in physical activities [47]. Al-Hazzaa et al. [35] conducted a similar study to that of Rideout et al. [46] on Saudi adolescents aged between 15 and 19 years of age to investigate the association of dietary habits with PA and technology use. Al-Hazzaa et al. [35] reported that excessive screen-time use was associated with significantly higher consumption of unhealthy food and inactivity.

The current study also showed that children who spend ≤5 h on their technology devices had a higher level of physical activity, and those who spend ≥6 h tend to have a lower level of physical activity. In contrast to our study, one study found that spending more than 2 h daily on screen-time had no association with lower levels of physical activity [26]. Karaca, et al. [32] examined the screen-time of adolescents by gender, school type, and sport participation using the household activities and sport indexes of the Physical Activity Assessment Questionnaire. They found that screen-time does not affect participation in physical activity. However, gender and school type (public or private) had a direct effect, as male students and those attending private schools had a higher rate of screen time than their counterparts [32]. Their results were linked to the customs and traditions of the country and to the socioeconomic class of the students. Females are more engaged with household work than males, and students of a high socioeconomic class have more access to technology at home than students with of a low socioeconomic class [32]. There has been no study until now that has compared the effect of technology use on PA between students in public and private Saudi schools. However, one can expect it to be high in students in private schools, and that can be attributed not only to the socioeconomic class of the students, but also to the schools’ financial resources. Private schools in Saudi Arabia are generally more technologically advanced than public schools, which might contribute to reduction in PA level of students in the latter.

Previous studies have shown that technology use is associated with a sedentary lifestyle, which can lead to several risk factors, the most prominent being obesity [48,49]. These studies found a significant relationship between increased body mass index (BMI)/obesity and sedentary behaviors such as watching television, computer use, and video games [20,50,51]. High screen use was recently found to be significantly associated with lower physical fitness [52]. Children exceeding screen time recommendations (at least 60 min of moderate to vigorous PA per day [53]) are at 60% high risk of having poor or very poor fitness level [52]. Tremblay et al. [54] found an inverse relationship between high sedentary behavior time and lower cardiorespiratory fitness, musculoskeletal fitness, and maximal oxygen uptake (VO_2max_). Furthermore, Pardee et al. [55], found that the chances of having metabolic syndrome in children with ≥5 h/day of screen-time were three times higher than in those with ≤1 h/day. Children with 4 h/day of screen-time were 3.3 times more likely to have hypertension than their counterparts with <2 h/day.

Participating in outdoor physical activity is one of the most important elements of an active lifestyle [56]. However, in Saudi Arabia, there are many factors that could limit participation in physical activity, such as customs and traditions, lack of local facilities, security, and road traffic density [57,58,59]. All these and other factors can be investigated in future studies.

### 4.2. Association between Socio-Demographics and Technology Use

The association between socio-demographics and technology time consumption among children was tested in the present study. No significant association between parental age and children’s use of technology use was found; however, mixed results have been reported previously. Some studies revealed that children of younger parents are more prone to using technology [25,60]. Other studies found no association between parental age and the child’s technology use [22,24,61]. The current study also showed no significant association between parental educational level or marital status and technology use. However, Carson and Janssen [20] found a negative association between parental educational level and use of technology, as higher technology use was found among children of parents with lower educational levels [62]. Furthermore, there was a significant correlation between marital status and technology use. A few studies have reported that children living with a single parent are more likely to use technology compared to children living in families with both parents [30,63,64].

This present study showed that family monthly income had an effect on the child’s technology use, similar to the findings of other studies [21,65]. Some studies have reported a positive association between family monthly income and child’s technology use, indicating that children of high-income families use technology more than those of low-income families, due to the reported ownership of devices [21,65]. On the other hand, other studies reported no association [33,61], or a negative association between family income and the child’s use of technology [60]. Several studies have investigated the relationship between ownership of devices and screen time consumption among children [22,31,62,66,67]. For instance, Alturki et al. [68] conducted a multicenter, cross-sectional study in Riyadh, Saudi Arabia on 1023 children between 9–12 years of age to investigate the relationship between ownership and duration spent on various electronic screen devices and association with the child’s weight status [68].They found a significant relation between device ownership and screen time, which was positively associated with the occurrence of obesity [68]. These studies are consistent with the current findings, as children who own their devices were prone to spend more time using technology. This result could be explained by the fact that ownership of a device leads to easy access to games, programs, and applications [22].

In contrast to the current study, significant differences between boys and girls have been observed in some studies regarding technology use. Some studies showed that girls spend more time watching television than boys [25,27]. Al-Hazzaa et al. [35] reported that technology use was significantly higher among Saudi female students than their counterparts; additionally, they were significantly less active and more sedentary. On the other hand, one study conducted in the USA [69] revealed that boys spend more time using technology than girls. Pyper et al. [70] and Dolatabadi et al. [71] found significantly more time spent in video game playing and computer/laptop use among boys than girls. In the Middle East, the tendency of boys to use technology more than girls might be attributed to culture and tradition, as the female role in the family revolves generally around doing household chores and helping family members. This could also be related to the skills required for using technology and different perspective regarding technology use between genders [71].

This study found a significant relationship between technology consumption time and the child’s age. Other studies found that older children are more likely to spend more time using technology than younger children [21,60,72]. This association may be attributed to the fact that older children are more skilled in using technology and able to access different types of technology [20]. However, Roberts et al. [73] reported that, on average, children aged between 8–10, 10–14, and 15–18 years spend 65, 52, and 33 min, respectively, on computer games per day. Roberts et al. attributed the increase in time spent on computer games to rapid advancements in communication and entertainment technologies and the growing use of handheld devices for game playing [73].

## 5. Conclusions

In conclusion, this study showed that high use of technology was significantly associated with low level of activity. Children who spend <5 h each week on their devices tend to have higher levels of physical activity compared to those who spend >6 h. Ownership of a device was significantly associated with higher technology time consumption. Furthermore, age of the child, educational level of the parents, screen time use, and owning electrical devices significantly predicted the level of physical activity among children of sampled parents. Thus, parental involvement is needed to reduce the time spent on screens, which can have a high impact on a child’s physical activity level.

### 5.1. Implications and Recommendations

Since children spend most of their time at school, the Ministry of Education should provide various sports and activity classes that enable children to participate in physical activity based on their preferred sports. The Ministry of Education should also work on improving the outdoor areas of schools, as some are poorly equipped and not designed for sports activities. Well-organized partnerships should be established between the Ministry of Education, Ministry of Health, Ministry of Municipalities, and private investors to design and invest in more appropriate places for children to practice their sports and to support the promotion of physical activity among children. Collaboration between the Ministry of Health and the Ministry of Information is recommended to educate families about the side effects of technology overuse. Furthermore, software developers need to be supported to design more active video games. Parents should be educated about the side effects of overuse of technology and encouraged to apply their role in controlling and limiting a child’s screen time and access to the internet. Parents are advised to increase outdoor activities and promote participation in housework.

### 5.2. Limitations

The used online questionnaire is a parent-reported questionnaire; therefore, we might have unintentionally missed parents with low literacy level, low technical abilities, with busy schedules, or who did not have access to computers or the Internet. Some of the questionnaire components were too long and resulted in incomplete answers, which were excluded later.

### 5.3. Future Research

Our study was limited to the central region of Saudi Arabia. Due to variation in sociodemographic variables between regions in KSA, future studies should be conducted to assess the relationship between physical activities and sociodemographic data and technology use between regions. The opinions of children on their abilities in the activities they engage in needs further investigation. Furthermore, using objective tools to measure the effect of technology use on physical activity rather than relying on self-reported data might help in developing more effective and cost-effective interventions.

### 5.4. Key Messages

- Daily technology use of devices by children is rising.
- Daily technology use of devices by children might lead to an inadequate level of physical activity.
- Children who spend <5 h each week on their devices tend to have higher levels of physical activity compared to those who spend >6 h.
- Parental involvement is needed to reduce the time children spend on screens, which can have a high impact on a child’s physical activity level.
- Inadequate physical activity among children may be influenced by the educational level of the parents.

## Figures and Tables

**Table 1:** Association between technology use and demographic data.

Variables	Screen-Time ≤5 h	Screen-Time ≥6 h	Used Test	
	*n* (%) 258 (56.33%)	*n* (%) 200 (43.67%)	*p*-Value	Value
Marital status **				
Married	239 (92.6)	179 (89.5)	0.05 *	12.69
Widowed	12 (4.7)	4 (2.0)		
Divorced	3 (1.2)	14 (7.0)		
Separated	4 (1.6)	3 (1.5)		
Educational level **				
Not educated	1 (0.4)	1 (0.5)		
Under high school	1 (0.4)	7 (3.5)	0.074	17.56
High school	21 (8.1)	31 (15.5)		
Bachelor	196 (76.0)	121 (60.5)		
Postgraduate	39 (15.1)	40 (40.0)		
Age group of parents (years)				
25–30	27 (10.5)	17 (8.5)		
30–35	145 (56.2)	111 (55.0)	0.34	3.34
35–40	79 (30.6)	60 (30.0)		
More than 40	7 (2.7)	12 (6.0)		
Income (SAR)				
Less than 5000	20 (7.8)	2 (1.0)	0.001 *	18.3
5000–10,000	34 (13.2)	40 (20.0)		
10,000–15,000	78 (30.2)	73 (36.5)		
15,000–20,000	57 (22.1)	30 (15.0)		
More than 20,000	69 (26.7)	55 (27.5)		
Sex (of child)				
Male	136 (52.7)	108 (54.0)		
Female	122 (47.3)	92 (46.0)	0.78	0.075
Child’s age (years)				
6	85 (32.9)	42 (21.0)	0.000 *	20.19
7	29 (11.2)	14 (7.0)		
8	51 (19.8)	42 (21.0)		
9	19 (7.4)	18 (9.0)		
10	38 (14.7)	31 (15.5)		
11	17 (6.6)	16 (8.0)		
12	19 (7.4)	37 (18.5)		
Does the child have his/her own device?				
Yes	190 (73.6)	186 (93.0)	0.000 *	28.71
No	68 (26.4)	14 (7.0)		
Child’s age when owned device **				
1	1 (0.4)	0 (0.0)		
2	4 (1.6)	6 (3.0)	0.75	24.92
3	17 (6.6)	21 (10.5)		
4	62 (24.0)	34 (17.0)		
5	45 (17.4)	24 (12.0)		
6	48 (18.6)	44 (22.0)		
7	22 (8.5)	25 (12.5)		
8	33 (12.8)	41 (20.5)		
9	11 (4.3)	2 (1.0)		
10	4 (1.6)	1 (0.5)		

* Note: *p*-value, statistically significant at ≤0.05; % = percentage. ** Fisher’s exact test was used—Otherwise a chi-squared test was used. Abbreviation: *n* = number of participants.

**Table 2:** Correlation between screen-time use and level of physical activity.

Correlation		Screen Time	High Level	Moderate Level	Low Level
Screen-time	Pearson Correlation	1	−0.138 *	−0.130	0.136
	Sig. (2-tailed)		0.047	0.062	0.051
High level	Pearson Correlation	−0.138 *	1	0.458 **	0.298 **
	Sig. (2-tailed)	0.047		0.000	0.000
Moderate level	Pearson Correlation	−0.130	0.458 **	1	0.377 **
	Sig. (2-tailed)	0.062	0.000		0.000
Low level	Pearson Correlation	0.136	0.298 **	0.377 **	1
	Sig. (2-tailed)	0.051	0.000	0.000	

* Correlation is significant at the 0.05 level (2-tailed). ** Correlation is significant at the 0.01 level (2-tailed).

**Table 3:** Predictors of low level physical activity level among children of sampled participants.

Variable	^‡^ B	** SE	^ Β	^^ *p* Value	95.0% Confidence Interval for B	
					Lower Bound	Upper Bound
Gender of the child	−0.226	0.211	0.798	0.284	0.528	1.206
Age group of the parents	−0.188	0.233	0.829	0.419	0.525	1.308
Marital status	−0.563	0.354	0.570	0.111	0.285	1.139
Educational level	−1.827	0.881	0.161	0.038 ^^^^	0.029	0.904
Family income	−0.234	0.247	0.791	0.343	0.488	1.284
Screen time use	−0.525	0.204	0.592	0.010 ^^^^	0.396	0.883
Own electronic device	0.009	0.270	0.250	0.000 ^^^^	0.132	0.472
Child age when owned device	0.430	0.227	1.537	0.041 ^^^^	1.984	2.401

^‡^ B is the regression coefficient/ unstandardized beta, ** SE is the Standard Error, ^ B is the standardized beta, ^^ *p* ≤ 0.05 is significant.
